# Impact of nasal and inhaled corticosteroids on SARS-CoV-2 infection susceptibility

**DOI:** 10.1016/j.jaci.2025.07.006

**Published:** 2025-07-21

**Authors:** Christian Rosas-Salazar, Tebeb Gebretsadik, Max A. Seibold, Camille M. Moore, Samuel J. Arbes, Leonard B. Bacharier, Steven M. Brunwasser, Carlos A. Camargo, William D. Dupont, Glenn T. Furuta, Rebecca S. Gruchalla, Ruchi S. Gupta, Daniel J. Jackson, Christine C. Johnson, Meyer Kattan, Gurjit K. Khurana Hershey, Andrew H. Liu, George T. O’Connor, Wanda Phipatanakul, Sima K. Ramratnam, Marc E. Rothenberg, Satria P. Sajuthi, Joshua Sanders, Christine M. Seroogy, Brittney M. Snyder, Lia Stelzig, Stephen J. Teach, Edward M. Zoratti, Alkis Togias, Patricia C. Fulkerson, Tina V. Hartert

**Affiliations:** a Department of Pediatrics, Biostatistics, and Medicine, Vanderbilt University Medical Center, Nashville; b Center for Genes, Environment and Health, National Jewish Health, Denver; c Rho, Inc, Chapel Hill; d Department of Psychology, Rowan University, Glassboro; e Department of Emergency Medicine and Pediatrics, Massachusetts General Hospital, Harvard Medical School, Boston; f Department of Pediatrics, University of Colorado School of Medicine, Aurora; g Department of Internal Medicine, University of Texas Southwestern Medical Center, Dallas; h Department of Pediatrics and Medicine, Northwestern University, Chicago; i Department of Pediatrics, University of Wisconsin-Madison, Madison; j Department of Public Health Sciences, Henry Ford Health System, Detroit; k Department of Pediatrics, Columbia University, New York; l Department of Pediatrics, Cincinnati Children’s Hospital Medical Center, University of Cincinnati College of Medicine, Cincinnati; m Department of Pulmonology, Allergy, Sleep, and Critical Care Medicine, Boston University, Boston; n Department of Pediatrics, George Washington University, Washington, DC; o National Institute of Allergy and Infectious Diseases, Rockville

**Keywords:** Airway, allergic rhinitis, asthma, COVID-19, inhaled corticosteroids, nasal corticosteroids, SARS-CoV-2

## Abstract

**Background:**

It is unknown whether nasal corticosteroid (NCS) or inhaled corticosteroid (ICS) use impacts the susceptibility to severe acute respiratory syndrome coronavirus 2 (SARS-CoV-2) infection.

**Objectives:**

We sought to examine the associations of NCS and ICS use with the risk of SARS-CoV-2 infection among individuals with allergic rhinitis or asthma.

**Methods:**

This is a prospective, multicenter, SARS-CoV-2 surveillance study of households with children. Nasal swabs were obtained from participants every 2 weeks with additional collections based on coronavirus disease 2019–related symptoms. In our primary adjusted models, we examined the association of NCS or ICS use at study entry (in participants with allergic rhinitis or asthma, respectively) with the time to the first SARS-CoV-2–positive quantitative PCR testing using Cox proportional hazard regression.

**Results:**

There were 2211 participants in the 1113 households included. The associations of NCS and ICS use with the risk of SARS-CoV-2 infection were modified by age (*P* for both interactions <.05). NCS and ICS use were individually associated with higher risks of SARS-CoV-2 infection among adults (adjusted hazard ratio [aHR] = 1.88, 95% CI: 1.14-3.12, *P* = .01, and aHR = 2.15, 95% CI: 1.003-4.63, *P* = .049, respectively). The association of NCS use with the risk of SARS-CoV-2 infection in adults was consistent in a series of sensitivity analyses. There was no association of NCS or ICS use with the risk of SARS-CoV-2 infection in children.

**Conclusion:**

Our findings suggest that the risk of SARS-CoV-2 infection is increased in adults who use NCS but not in children. Similar, albeit less consistent, age-dependent findings were observed for ICS use. While the results of this observational study should be interpreted with caution, they emphasize the need to conduct studies to understand potential mechanisms that could explain these findings.

The upper respiratory tract (URT) is typically the site of primary infection of severe acute respiratory syndrome coronavirus 2 (SARS-CoV-2), the respiratory virus responsible for the coronavirus disease 2019 (COVID-19) pandemic.^1,2^ Following airborne exposure to SARS-CoV-2, certain host factors (such as differences in the expression of cell receptors, intracellular signaling, and other host defense pathways) can determine the effective entry of SARS- CoV-2 into the URT cells and the establishment of infection.^2–4^

Topical airway corticosteroids (such as nasal corticosteroids [NCS] or inhaled corticosteroids [ICS]) are the mainstay of treatment for the long-term management of allergic rhinitis and asthma.^5,6^ These commonly used medications may influence the SARS-CoV-2 URT cell entry process and acute immune response,^4,7–11^ leading us to hypothesize that the risk of SARS-CoV-2 infection differs among individuals who use topical airway corticosteroids when compared to those who do not use these medications. This is one of the hypotheses in the design of the HEROS (Human Epidemiology and RespOnse to SARS-CoV-2 Study), a prospective, multicenter, SARS-CoV-2 surveillance study of households enriched for children and adults with allergic rhinitis and asthma.

## METHODS

### Overview of the study population

The HEROS study population included households with children (ages <21 years) participating in 20 National Institutes of Health–funded cohorts focused on atopic diseases in 12 US cities between May 2020 and February 2021. Enrollment in HEROS required the participation of the cohort-participating child and 1 primary adult caregiver living in the same household. Two other household members (1 child and 1 other adult caregiver) could also enroll. To minimize confounding by indication, we restricted the study population to participants who self-reported a prior health care provider diagnosis of allergic rhinitis (for the analyses of NCS use) or asthma (for the analyses of ICS use). Participants were followed for at least 24 weeks and up to 28 weeks after enrollment. The detailed methods for HEROS have been previously reported.^12,13^ The US Department of Health and Human Services Office for Human Research Protections and the Institutional Review Boards of all participating institutions deemed the study a public health surveillance study. Primary adult caregivers were provided an online study information fact sheet, which was a consent-like document containing components of the US Federal Policy for the Protection of Human Subjects. To enroll, primary adult caregivers were required to agree that they read, understood, and reviewed this online study information fact sheet with all participating household members. This sheet included an option to select having the study site contact the primary adult caregiver to answer any questions.

### Study questionnaires and NCS and ICS ascertainment

Primary adult caregivers completed study entry and end-of-study questionnaires that collected comprehensive information on sociodemographic factors, current and past medical history (including a prior health care provider diagnosis of allergic rhinitis or asthma), and current use of medications (including use of topical airway corticosteroids). These questionnaires were primarily developed from previously validated instruments (including selected items from the International Study of Asthma and Allergies in Childhood questionnaire^14^) and were used to ascertain self-report use of NCS and ICS at study entry and end of study. In addition, primary adult caregivers completed biweekly questionnaires to capture potential exposures to SARS-CoV-2 and weekly questionnaires to capture symptoms related to COVID-19. Similar to the 2020 US Census Operational Plan, primary adult caregivers completed each questionnaire on behalf of all household members.^15^ The questionnaires were completed remotely via online platforms, short text messages, or telephone communications with the study site staff.

### SARS-CoV-2 testing

Self- or caregiver-collected nasal swabs were obtained from participants every 2 weeks between May 2020 and February 2021, which was the COVID-19 pandemic period almost entirely before SARS-CoV-2 vaccine availability. In addition, participants completed weekly symptom questionnaires. If any household member developed symptoms related to COVID-19 during this time, a prespecified algorithm prompted an additional self or caregiver nasal swab collection from all participants. SARS-CoV-2 quantitative PCR testing was conducted in all nasal swabs collected using the US Centers for Disease Control and Prevention SARS-CoV-2 N1/N2 and ribonuclease P housekeeping gene assays as previously described.^12,13^

### Blood allergen-specific IgE testing

Self- or caregiver-collected capillary blood samples were obtained from participants ages >2 years at least once during the study using the Tasso home collection device (Tasso Inc, Seattle, Wash). Multiplex specific IgE testing to 112 aeroallergens and foods from 48 different sources was conducted in the capillary blood samples collected using the ImmunoCAP ISAAC assay (Phadia AB, Portage, Mich).

### Statistical analyses

Descriptive statistics are presented as median (interquartile range [IQR]) for continuous variables and frequency (percentage) for categorical variables. We examined the association of NCS or ICS use at study entry with the time to the first SARS-CoV-2–positive quantitative PCR testing by plotting Kaplan-Meier cumulative morbidity curves and by conducting Cox proportional hazard regression with a robust sandwich covariance estimate to account for clustering of participants within a household. The assumption of proportionality of the primary exposure variables was assessed using a global test based on Schoenfeld residuals and visual residual plots checking. Because there was no clear violation of the proportional hazards assumption, we assumed proportional hazards for all variables. To account for geographic differences in SARS-CoV-2 infection risk over time, baseline hazards were stratified by study site. The primary adjusted models included the participant’s age (as a nonlinear term with restricted cubic splines), sex, race and ethnicity (categorized as non-Hispanic White vs other), and body mass index percentile^16^ as *a priori* selected covariates. We also tested for interactions between NCS or ICS use at study entry and age, sex, and race and ethnicity, separately by adding cross-product terms to the primary adjusted models.

In supplementary analyses, we built secondary adjusted models by adding the following covariates to the primary adjusted models: (1) the area deprivation index (a marker of socioeconomic disadvantage at the neighborhood level that incorporates theoretical domains of income, education, employment, and housing quality),^17^ (2) use of other topical airway corticosteroids at study entry, (3) a modified comorbidity index (based on the Charlson comorbidity index [Table E1 in this article’s Online Repository at www.jacionline.org]^18^), (4) current smoking at study entry, (5) a prior health care provider diagnosis of diabetes type 1 or 2, and (6) use of blood pressure medications (such as angiotensin-converting enzyme [ACE] inhibitors or angiotensin II receptor blockers) at study entry.

To assess whether misclassification of the exposure or the selection of the study population could have impacted our results, we conducted sensitivity analyses by only including participants who had data on NCS and ICS use at both study entry and end of study, as well as only including participants with allergic rhinitis who had evidence of allergen sensitization by blood allergen-specific IgE testing. Likewise, we evaluated the specificity of the associations and potential confounding by the underlying disease severity through sensitivity analyses examining (1) the association of use of other allergic rhinitis medications (eg, leukotriene receptor antagonists, nasal antihistamines, or oral antihistamines) or other asthma controllers (eg, leukotriene receptor antagonists, long-acting muscarinic antagonists, or biologic therapies) at study entry with the risk of SARS-CoV-2 infection, and (2) the relationship between the number of allergic rhinitis medications or asthma controllers used at study entry (as markers of allergic rhinitis and asthma severity, respectively) and the risk of SARS-CoV-2 infection.

Statistical analyses used 2-sided *P* values and significance was defined as *P* < .05. Statistical analyses were performed using R version 4.0.1 (R Foundation, Vienna, Austria).^19^

## RESULTS

### Baseline characteristics of the study population

There were 4142 participants from 1394 households enrolled in HEROS who contributed at least 1 nasal swab between May 2020 and February 2021. The baseline characteristics of these 4142 participants have been described elsewhere.^13^ Of these 4142 participants, 2211 participants (53%) (age range: 0-80 years) from 1113 households had a prior health care provider diagnosis of allergic rhinitis (n = 1892 [46%]) or asthma (n = 1150 [28%]) and comprised the current study population. Among these 2211 participants there were 1164 children (53%, median age 12 [IQR: 7-15] years) and 1047 adults (47%, median age 41 [IQR: 36-47] years). The majority of these 2211 participants were female (1285 [58%]) and non-Hispanic White (1220 [57%]) (Table I). Of the 2211 participants, 145 (7%) developed a SARS-CoV-2 infection during the study period.

In comparison to participants with allergic rhinitis who were not using NCS at study entry, those who were using NCS at study entry were more likely to be of a race and ethnicity other than non-Hispanic White, ever had asthma, and be using ICS at study entry, and less likely to be currently smoking at study entry (Table I). Participants with asthma who were using ICS at study entry were younger and more likely to be of a race and ethnicity other than non-Hispanic White, be using NCS at study entry, and ever had allergic rhinitis than those who were not using ICS at study entry (Table I).

### Effect modification by participant’s age

**FIG 1.**
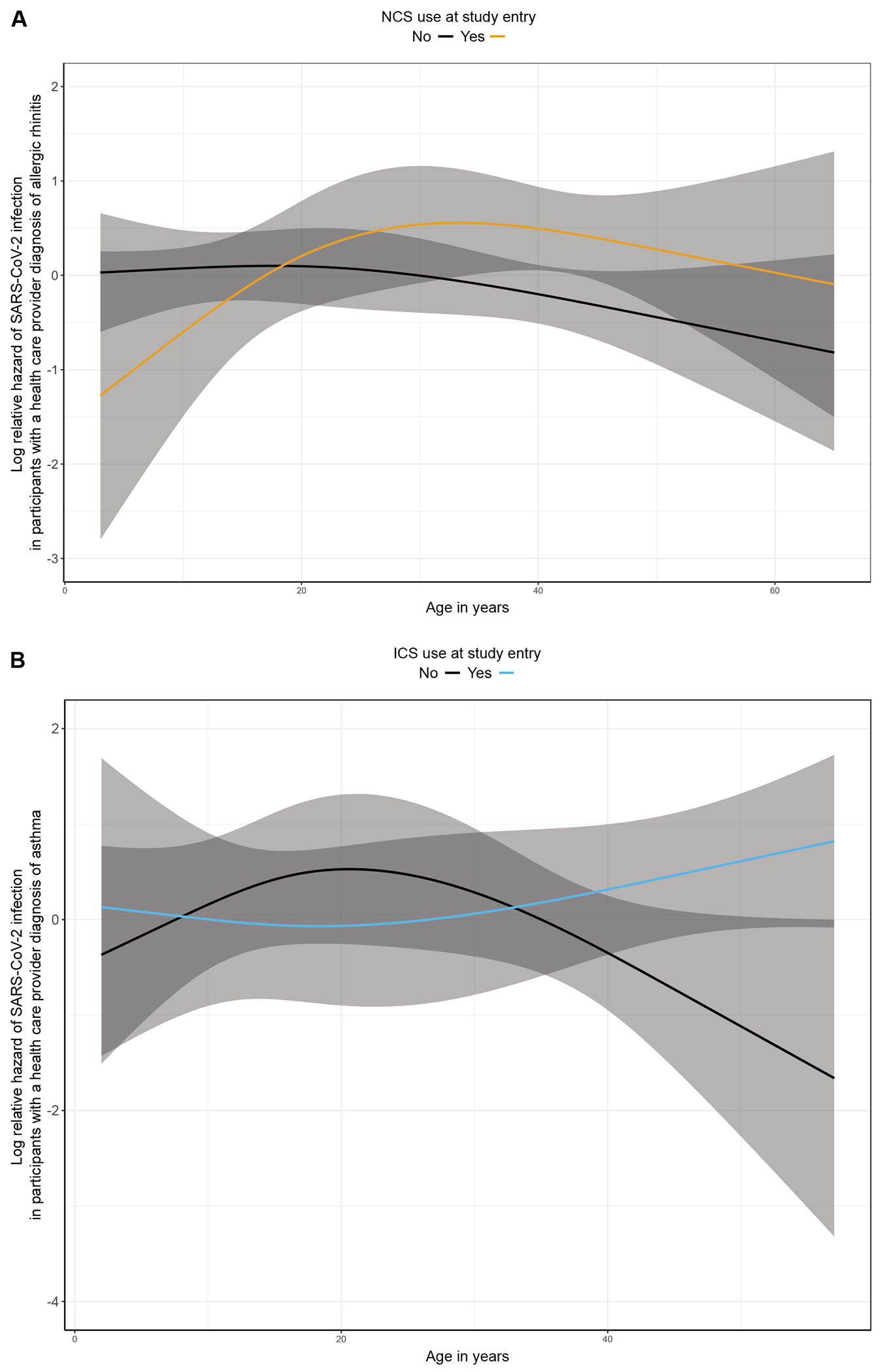
Effect modification of age on the association of using topical airway corticosteroids (NCS and ICS) with the risk of SARS-CoV-2 infection. **(A)** Log relative hazard of SARS-CoV-2 infection among participants with a prior health care provider diagnosis of allergic rhinitis by age and NCS use at study entry. **(B)** Log relative hazard of SARS-CoV-2 infection among participants with a prior health care provider diagnosis of asthma by age and ICS use at study entry.

In participants with allergic rhinitis, there was a statistically significant effect modification of the association of NCS use at study entry with the risk of SARS-CoV-2 infection by age (*P* for interaction = .02) (Fig 1, A). Similarly, the effect of ICS use at study entry on the risk of SARS-CoV-2 infection among participants with asthma was modified by age (*P* for interaction = .047) (Fig 1, B). There was no statistically significant effect modification of sex or race and ethnicity on the associations of NCS or ICS use at study entry with the risk of SARS-CoV-2 infection among participants with allergic rhinitis or asthma, respectively (*P* > .05 for all interaction terms). Based on these results, subsequent statistical analyses were conducted separately in children (<21 years of age) and adults (≥21 years of age).

### Use of NCS in participants with allergic rhinitis and the risk of SARS-CoV-2 infection

**FIG 2.**
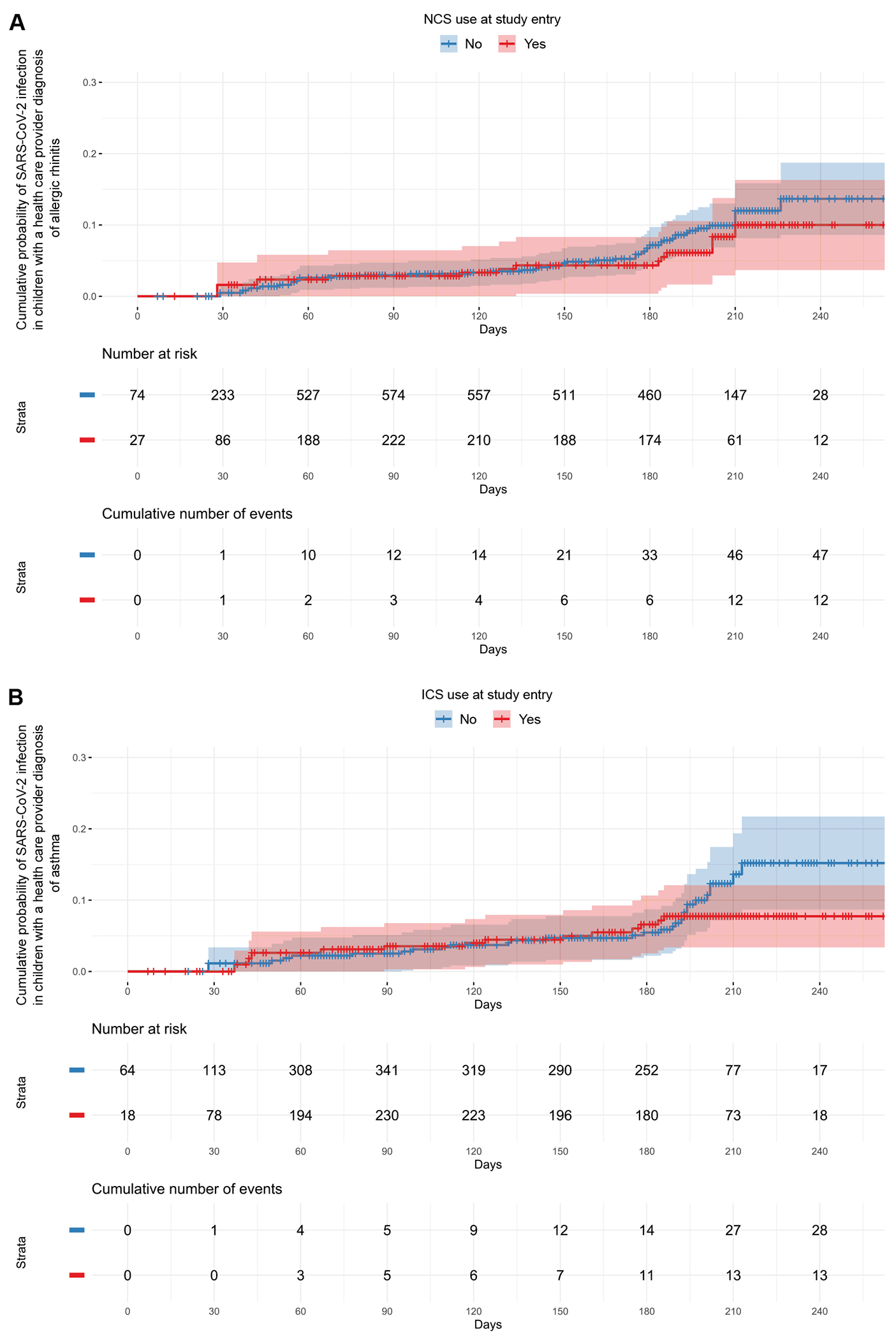
Risk of SARS-CoV-2 infection among children by use of topical airway corticosteroids (NCS and ICS). **(A)** Kaplan-Meier curve of the cumulative probability of SARS-CoV-2 infection among children with a prior health care provider diagnosis of allergic rhinitis by NCS use at study entry. **(B)** Kaplan-Meier curve of the cumulative probability of SARS-CoV-2 infection among children with a prior health care provider diagnosis of asthma by ICS use at study entry. The x-axis represents the time from the start of the study to the first SARS-CoV-2–positive quantitative PCR testing or the last available nasal swab in days.

**FIG 3.**
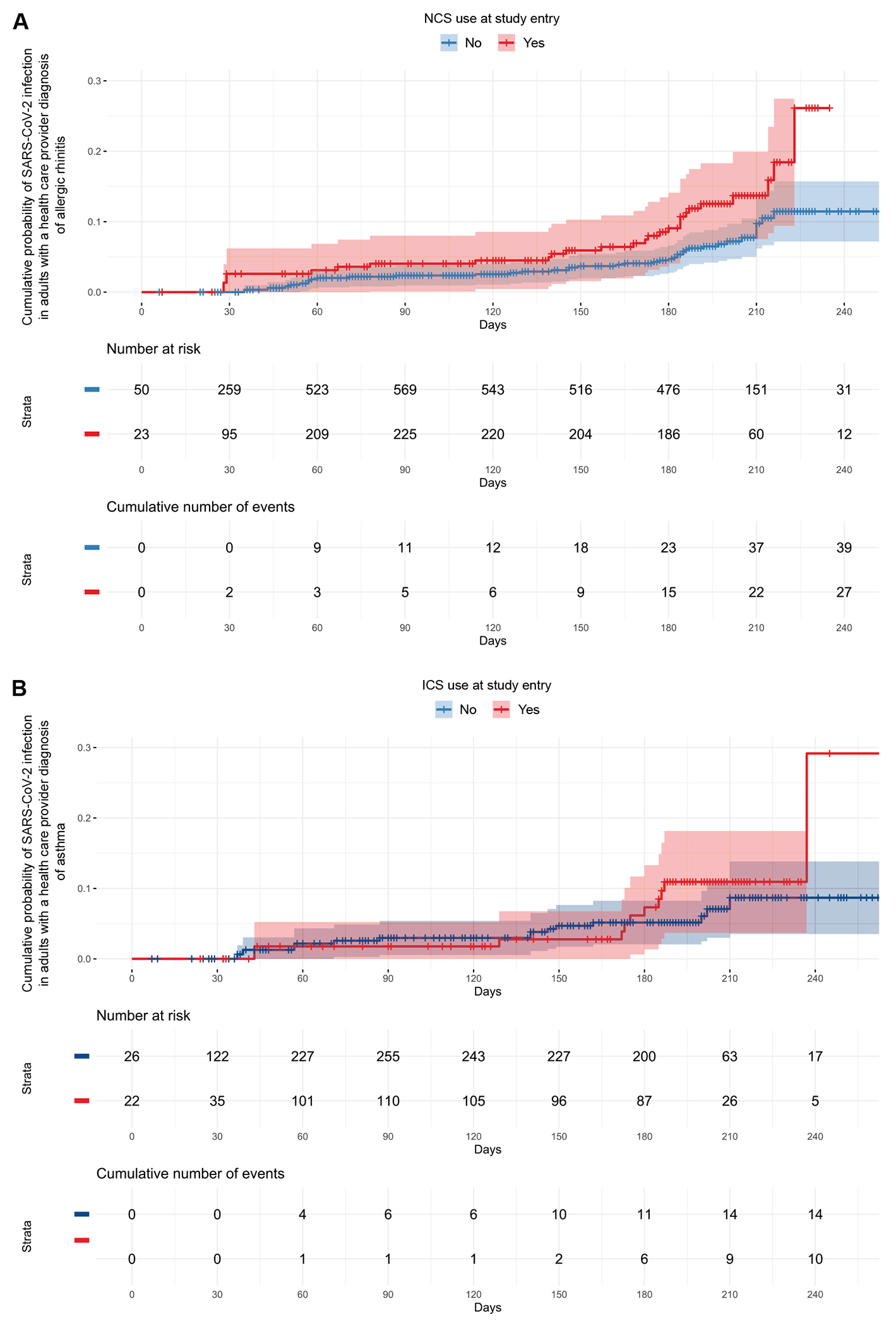
Risk of SARS-CoV-2 infection among adults by use of topical airway corticosteroids (NCS and ICS). **(A)** Kaplan-Meier curve of the cumulative probability of SARS-CoV-2 infection among adults with a prior health care provider diagnosis of allergic rhinitis by NCS use at study entry. **(B)** Kaplan-Meier curve of the cumulative probability of SARS-CoV-2 infection among adults with a prior health care provider diagnosis of asthma by ICS use at study entry. The x-axis represents the time from the start of the study to the first SARS-CoV-2–positive quantitative PCR testing or the last available nasal swab in days.

NCS use at study entry was not associated with the risk of SARS-CoV-2 infection among children with allergic rhinitis in our primary adjusted model (adjusted hazard ratio [aHR] = 0.65, 95% CI:0.27-1.57, *P* = .3) (Table II and Fig 2, A). In contrast, adults with allergic rhinitis who were using NCS at study entry had ~90% higher risk of SARS-CoV-2 infection than those who were not using NCS in our primary adjusted model (aHR = 1.88, 95% CI: 1.14-3.12, *P* = .01) (Table III and Fig 3, A). In adults with allergic rhinitis, the effect size and statistical significance of the association remained consistent in secondary adjusted models that included the area deprivation index, ICS use at study entry, the modified comorbidity index, current smoking at study entry, a prior health care provider diagnosis of diabetes type 1 or 2, and use of blood pressure medications at study entry as additional covariates (Table III).

In sensitivity analyses, the risk of SARS-CoV-2 infection remained increased among adults with allergic rhinitis using NCS at both study entry and end of study compared to those not using these medications at any time point (Table E2 in this article’s Online Repository at www.jacionline.org). Restricting analyses to the subgroup of adults with allergic rhinitis who had evidence of allergen sensitization by blood allergen-specific IgE testing revealed similar directionality and effect size, although this reduced the size of the study population and the association was not statistically significant (Table E3 in this article’s Online Repository at www.jacionline.org). Use of medications for allergic rhinitis other than NCS or the number of allergic rhinitis medications used (a marker of allergic rhinitis severity) at study entry was not associated with the risk of SARS-CoV-2 infection among adults with allergic rhinitis (Tables E4 and E5 in this article’s Online Repository at www.jacionline.org).

### Use of ICS in participants with asthma and the risk of SARS-CoV-2 infection

There was no association of ICS use at study entry with the risk of SARS-CoV-2 infection among children with asthma in our primary adjusted model (aHR = 0.86, 95% CI = 0.40-1.87, *P* = .7) (Table II and Fig 2, B). Similar results were obtained when restricting analyses to the subgroup of children with asthma ages 5 years and older (Table E6 in this article’s Online Repository at www.jacionline.org). In contrast, adults with asthma who were using ICS at study entry had ~2-fold higher risk of SARS-CoV-2 infection than those who were not using ICS in our primary adjusted model (aHR = 2.15, 95% CI: 1.003-4.63, *P* = .049) (Table III and Fig 3, B). In adults with asthma, the effect size and statistical significance of this association remained consistent in secondary adjusted models that included ICS use at study entry, the modified comorbidity index, and use of blood pressure medications at study entry as additional covariates (Table III). Likewise, the effect size of this association remained similar in secondary adjusted models that included the area deprivation index, current smoking at study entry, and a prior health care provider diagnosis of diabetes type 1 or 2 as additional covariates but the association was not statistically significant (Table III).

The risk of SARS-CoV-2 infection was not increased among adults with asthma when ascertaining ICS use at both study entry and end of study (Table E7 in this article’s Online Repository at www.jacionline.org). Use of asthma controllers other than ICS at study entry was not associated with the risk of SARS-CoV-2 infection among adults with asthma (Table E8 in this article’s Online Repository at www.jacionline.org). However, the number of asthma controllers used (a marker of asthma severity) at study entry was positively associated with the risk of SARS-CoV-2 infection in this age group (Table E9 in this article’s Online Repository at www.jacionline.org).

In adults with allergic rhinitis or asthma, the combined use of NCS and ICS at study entry did not increase the risk of SARS-CoV-2 infection. However, adults with allergic rhinitis or asthma using only NCS at study entry had a risk of SARS-CoV-2 infection that was intermediate between the risk of those using only ICS at study entry and that of those using both NCS and ICS at study entry (Table E10 in this article’s Online Repository at www.jacionline.org).

## DISCUSSION

Our findings suggest that the risk of SARS-CoV-2 infection is increased in adults who use NCS but not in children. Similar, albeit less consistent, age-dependent findings were observed for ICS use. Our findings are important as topical airway corticosteroids are among the most commonly used medications for the management of pediatric and adult allergic and respiratory disorders,^5,6^ and understanding their safety profile could aid COVID-19 public health measures. These findings, while informative, are constrained by the observational study design and do not imply causation.

We can only hypothesize on the potential mechanisms underlying the association of NCS use and the risk of SARS-CoV-2 infection in adults. The entry of SARS-CoV-2 into the URT cells and eventually host infection starts with its binding to respiratory epithelial cell receptors (such as the ACE2 receptor), subsequent membrane fusion, and ultimately viral replication and release.^1,2^ In addition to blunting the local immune response, NCS can impact many of these steps and lead to major changes in the nasal microenvironment, which could explain our results.^1,9,10^ However, little is known about the relationship between NCS use and the SARS-CoV-2 URT cell entry process in humans. Interestingly, available *in vitro* data suggest that NCS could decrease the expression of ACE2 receptors in respiratory epithelial cells, as well as impair the SARS-CoV-2 replication process, which—in contrast to our findings—should lower the risk of SARS-CoV-2 infection.^20^ On the other hand, NCS use in atopic adults can decrease the levels of type-2 cytokines (such as IL-13) or eosinophils in the URT, which could then upregulate the expression of ACE2 receptors in respiratory epithelial cells and possibly increase the risk of SARS-CoV-2 infection.^10,21–26^ To our knowledge, while studies examining the effect of NCS use on COVID-19 morbidity in SARS-CoV-2–infected adults have had conflicting results,^27–30^ no other studies have examined the association of NCS use with the risk of SARS-CoV-2 infection in children or adults.

Our findings of a differential effect of NCS use on the risk of SARS-CoV-2 infection in children compared to adults are in line with described age-dependent effects of SARS-CoV-2 on several COVID-19 outcomes.^31,32^ For example, studies early in the COVID-19 pandemic suggested that children were less likely to be infected with SARS-CoV-2 (posited to be due in part to a lower expression of ACE2 receptors in respiratory epithelial cells compared to adults).^33,34^ However, in the HEROS study population, we previously demonstrated that the risk of SARS-CoV-2 infection does not differ between children and adults but rather children are substantially more likely to have asymptomatic or paucisymptomatic COVID-19 and thus clinically underrecognized SARS-CoV-2 infection.^13^

The risk of SARS-CoV-2 infection among adults using NCS remained increased after controlling for various potential confounders, but—as it is the case for all observational studies—our results could have been biased by confounding. To prevent confounding by indication in our analyses of NCS use, we only included participants with allergic rhinitis. However, NCS use can be related to the severity of allergic rhinitis, which may be acting as an additional confounder. To address this, we conducted a separate analysis to examine the association of the number of allergic rhinitis medications (a marker of underlying allergic rhinitis severity) with the risk of SARS-CoV-2 infection in adults and found no association. Yet, the independent effects of NCS use and the severity of allergic rhinitis on the risk of SARS-CoV-2 are difficult to disentangle and we lacked data on other markers of allergic rhinitis severity.

Some of our findings suggest that the risk of SARS-CoV-2 infection could also be increased in adults who use ICS. However, we found a relationship between the number of asthma controllers (a marker of the underlying asthma severity) and the risk of SARS-CoV-2 infection in adults. These findings suggest that confounding could have biased the association between ICS use and the risk of SARS-CoV-2 in adults, which prevents us from establishing firm conclusions about this relationship. Of note, it is certainly possible that only NCS (and not ICS) impact the risk of SARS-CoV-2 in adults, as the URT is the site of primary infection of SARS-CoV-2 and the first line of defense against COVID-19. The URT is not only the entry portal of the airways but also contains the highest percentage of ACE2-expressing respiratory epithelial cells across all the airways.^3,4^ In contrast to NCS, ICS are largely deposited in the more distal and lower airways, which are anatomical regions important in the severity of COVID-19 (an outcome for which ICS have demonstrated success in some but not all clinical trials^28,35,36^) but are likely less relevant for establishing SARS-CoV-2 infection.^1,4,37,38^

The strengths of our study include the prospective surveillance study design, the enrollment of participants across the US, the overall young population, and the careful ascertainment of the exposure and outcome of interest. We should also acknowledge some limitations in addition to confounding. First, it is possible that the effect of using topical airway corticosteroids on SARS-CoV-2 infection could depend on the underlying inflammatory phenotype, the SARS-CoV-2 strain, or on the potency, formulation, or pharmacodynamics of the specific drug, which we could not examine.^11,23,33^ Second, we may not have had sufficient statistical power to detect some associations. Third, there could have been misclassification of the exposure, as we lacked data on medication adherence, or misclassification of the outcome, based on the nasal sampling interval and missed biospecimens. Similarly, we defined allergic rhinitis based on self-report of a prior health care provider diagnosis, which could have led to misclassification of the study population. While we also conducted sensitivity analyses by only including participants with allergic rhinitis who had evidence of allergen sensitization by blood allergen-specific IgE testing, this led to a decreased sample size. Last, because the underlying atopic inflammation in persons with allergic rhinitis or asthma could impact SARS-CoV-2 URT cell entry process,^33^ our results may not be generalizable to those using NCS or ICS for other medical conditions. However, allergic rhinitis and asthma are the most common indications and account for the majority of use of topical airway corticosteroids in both the pediatric and adult population.

In summary, our results suggest the risk of SARS-CoV-2 infection is increased among adults who use NCS and potentially among those who use ICS when compared to those who do not use these medications, whereas we found no association of NCS or ICS use and SARS-CoV-2 infection in children. While the results of this observational study should be interpreted with caution and should not preclude the prescription of topical airway corticosteroids, they emphasize the need to conduct studies to understand potential mechanisms that could explain these findings and confirm whether the age-dependent relationship between NCS use and the risk of SARS-CoV-2 infection is causal. Follow-up studies are important for informing patient care, clinical guidelines, and COVID-19 public health measures.

## Supplementary Material

1

## Figures and Tables

**TABLE I.** Baseline characteristics of study participants included in statistical analyses*†

	NCS use at study entry among participants with a prior health care provider diagnosis of allergic rhinitis‡			ICS use at study entry among participants with a prior health care provider diagnosis of asthma‡			All (N = 2211)
	No (n = 1369)	Yes (n = 523)	All (n = 1892)	No (n = 736)	Yes (n = 414)	All (n = 1150)	
Age (y)	20 (12-41)	18 (12-42)	20 (12-41)	17 (12-38)	14 (10-34)	16 (11-37)	18 (12-40)
Age group							
Children (<21 y of age)	694 (51)	266 (51)	960 (51)	419 (57)	290 (70)	709 (62)	1164 (53)
Adults (≥21 y of age)	675 (49)	257 (49)	932 (49)	317 (43)	124 (30)	441 (38)	1047 (47)
Female sex	797 (58)	316 (60)	1113 (59)	422 (58)	225 (54)	647 (56)	1285 (58)
Race and ethnicity other than non-Hispanic White	491 (37)	242 (47)	733 (40)	374 (52)	274 (67)	648 (58)	938 (43)
Body mass index, percentile	84 (56-96)	84 (54-96)	84 (55-96)	86 (62-96)	87 (53-97)	86 (58-97)	85 (56-96)
Area deprivation index	43 (25-66)	41 (22-65)	42 (24-66)	41 (23-68)	40 (17-69)	41 (21-68)	42 (23-66)
Modified comorbidity index							
0	1156 (84)	448 (86)	1604 (85)	648 (88)	358 (86)	1006 (87)	1897 (86)
1	153 (11)	56 (11)	209 (11)	67 (9)	44 (11)	111 (10)	229 (10)
2	44 (3)	11 (2)	55 (3)	18 (2)	5 (1)	23 (2)	58 (3)
3	9 (1)	3 (1)	12 (1)	3 (0)	3 (1)	6 (1)	13 (1)
≥4	7 (1)	5 (1)	12 (1)	0 (0)	4 (1)	4 (0)	14 (1)
Current smoking at study entry§	61 (4)	12 (2)	73 (4)	18 (2)	13 (3)	31 (3)	85 (4)
Prior health care provider diagnosis of diabetes type 1 or 2	37 (3)	18 (3)	55 (3)	15 (2)	10 (2)	25 (2)	67 (3)
Use of blood pressure medication at study entry	84 (6)	42 (8)	126 (7)	39 (5)	24 (6)	63 (5)	141 (6)
Prior health care provider diagnosis of allergic rhinitis	1369 (100)	523 (100)	1892 (100)	508 (69)	323 (78)	831 (72)	1892 (86)
Prior health care provider diagnosis of asthma	538 (39)	293 (56)	831 (44)	736 (100)	414 (100)	1150 (100)	1150 (52)
NCS use at study entry	—	—	—	158 (21)	183 (44)	341 (30)	571 (26)
ICS use at study entry	171 (12)	168 (32)	339 (18)	—	—	—	430 (19)
Study site:							
Boston, Mass	155 (11)	55 (11)	210 (11)	116 (16)	70 (17)	186 (16)	270 (12)
Chicago, Ill	129 (9)	58 (11)	187 (10)	76 (10)	52 (13)	128 (11)	226 (10)
Cincinnati, Ohio	345 (25)	125 (24)	470 (25)	149 (20)	77 (19)	226 (20)	511 (23)
Dallas, Tex	12 (1)	17 (3)	29 (2)	11 (1)	17 (4)	28 (2)	33 (1)
Denver, Colo	72 (5)	33 (6)	105 (6)	41 (6)	44 (11)	85 (7)	126 (6)
Detroit, Mich	204 (15)	68 (13)	272 (14)	109 (15)	39 (9)	148 (13)	313 (14)
Madison, Wis	28 (2)	3 (1)	31 (2)	11 (1)	5 (1)	16 (1)	32 (1)
Marshfield, Wis	27 (2)	8 (2)	35 (2)	9 (1)	3 (1)	12 (1)	41 (2)
Nashville, Tenn	297 (22)	90 (17)	387 (20)	106 (14)	25 (6)	131 (11)	429 (19)
New York City, NY	65 (5)	36 (7)	101 (5)	69 (9)	50 (12)	119 (10)	142 (6)
St Louis, Mo	22 (2)	11 (2)	33 (2)	21 (3)	13 (3)	34 (3)	45 (2)
Washington, DC	13 (1)	19 (4)	32 (2)	17 (2)	19 (5)	36 (3)	42 (2)

* Data presented as median (IQR) for continuous variables or number (%) for categorical variables.

† Data calculated for participants with complete data.

‡ Only participants with a prior health care provider diagnosis of allergic rhinitis or asthma were included in statistical analyses.

§ Only captured in participants older than 15 years of age.

**TABLE II.** The association of using topical airway corticosteroids (NCS and ICS) with the risk of SARS-CoV-2 infection among children*

	Unadjusted	Adjusted†
NCS use at study entry in children with allergic rhinitis	0.64 (0.29-1.42), *P* = .3 (n = 960)	0.65 (0.27-1.57), *P* = .3 (n = 878)
ICS use at study entry in children with asthma	0.72 (0.35-1.50), *P* = .4 (n = 709)	0.86 (0.40-1.87), *P* = .7 (n = 634)

* Data presented as HR (95% CI) and *P* value. The total sample size with available data included in the model (n) is also shown. Estimates obtained from unadjusted and adjusted Cox regression models.

† The adjusted model includes age (as a nonlinear term with restricted cubic splines), sex, race and ethnicity (categorized as non-Hispanic White vs other), and body mass index percentile as covariates.

**TABLE III.** The association of using topical airway corticosteroids (NCS and ICS) with the risk of SARS-CoV-2 among adults*

	Unadjusted	Adjusted						
		Model 1†	Model 2‡	Model 3§	Model 4‖	Model 5¶	Model 6**	Model 7††
NCS use at study entry in adults with allergic rhinitis	1.80 (1.09-2.95), *P* = .02 (n = 932)	1.88 (1.14-3.12), *P* = .01 (n = 906)	1.73 (1.03-2.89), *P* = .04 (n = 890)	1.92 (1.12-3.28), *P* = .02 (n = 906)	1.87 (1.11-3.13), *P* = .02 (n = 906)	1.87 (1.13-3.12), *P* = .02 (n = 906)	1.92 (1.15-3.20), *P* = .01 (n = 906)	1.93 (1.17-3.18), *P* = .01 (n = 906)
ICS use at study entry in adults with asthma	1.85 (0.83-4.20), *P* = .1 (n = 441)	2.15 (1.003-4.63), *P* = .049 (n = 427)	2.15 (0.99-4.63), *P* = .05 (n = 421)	2.36 (1.06-5.25), *P* = .04 (n = 427)	2.21 (1.04-4.71), *P* = .04 (n = 427)	2.06 (1.00-4.42), *P* = .06 (n = 427)	2.14 (1.00-4.58), *P* = .05 (n = 427)	2.15 (1.003-4.62), *P* = .049 (n = 427)

* Data presented as HR (95% CI) and *P* value. The total sample size with available data included in the model (n) is also shown. Estimates obtained from unadjusted and adjusted Cox regression models.

† Adjusted model 1 includes age (as a nonlinear term with restricted cubic splines), sex, race and ethnicity (categorized as non-Hispanic White vs other), and body mass index percentile as covariates.

‡ Adjusted model 2 includes the same variables as model 1 plus the area deprivation index as an additional covariate.

§ Adjusted model 3 includes the same variables as model 1 plus ICS use at study entry (for the model of NCS use) or NCS use at study entry (for the model of ICS use) as an additional covariate.

‖ Adjusted model 4 includes the same variables as model 1 plus the modified comorbidity index as an additional covariate.

¶ Adjusted model 5 includes the same variables as model 1 plus current smoking at study entry as an additional covariate.

** Adjusted model 6 includes the same variables as model 1 plus a prior health care provider diagnosis of diabetes type 1 or 2 as an additional covariate.

†† Adjusted model 7 includes the same variables as model 1 plus use of blood pressure medications at study entry as an additional covariate.

## Clinical implications:

Our results suggest the risk of SARS-CoV-2 infection is increased among adults who use NCS. Follow-up studies are important for informing patient care, clinical guidelines, and COVID-19 public health measures.

## Abbreviations used

ACE: Angiotensin converting enzyme
aHR: Adjusted hazard ratio
COVID-19: Coronavirus disease 2019
ICS: Inhaled corticosteroids
IQR: Interquartile range
NCS: Nasal corticosteroids
SARS-CoV-2: Severe acute respiratory syndrome coronavirus 2
URT: Upper respiratory tract
