# Supplementary material for: Impact of nasal and inhaled corticosteroids on SARS-CoV-2 infection susceptibility
Source: J Allergy Clin Immunol. Author manuscript; Available in PMC 2025 Nov 8. (PMC12596024; doi:10.1016/j.jaci.2025.07.006)
Supplement: 1 [file NIHMS2121158-supplement-1.pdf]

TABLE E1. Modified comorbidity index among study participants

Risk factors	Points
Age, y	
<50	0
50-59	+1
60-69	+2
70-79	+3
≥80	+4
Prior health care provider diagnosis of heart attack	+1
Prior health care provider diagnosis of heart failure	+1
Prior health care provider diagnosis of peripheral vascular disease	+1
Prior health care provider diagnosis of chronic obstructive pulmonary disease or emphysema	+1
Prior health care provider diagnosis of autoimmune conditions	+1
Prior health care provider diagnosis of diabetes type 1 or 2	+1
Prior health care provider diagnosis of cancer	+2

TABLE E2. The association of NCS use at study entry and end of the study with the risk of SARS-CoV-2 infection among adults with a prior health care provider diagnosis of allergic rhinitis*

	Unadjusted (n = 622)	Adjusted (n = 606)†
NCS use at study entry and end of study	2.11 (1.11-4.02), *P* = .02	2.14 (1.13-4.04), *P* = .02

*Data presented as HR (95% CI) and *P* value. The total sample size with available data included in the model (n) is also shown. Estimates obtained from unadjusted and adjusted Cox regression models.

†The adjusted model includes age (as a nonlinear term with restricted cubic splines), sex, race and ethnicity (categorized as non-Hispanic White vs other), and body mass index percentile as covariates.

TABLE E3. The association of NCS use at study entry with the risk of SARS-CoV-2 infection among adults with a prior health care provider diagnosis of allergic rhinitis and evidence of allergen sensitization by a blood allergen-specific IgE testing*

	Unadjusted (n = 331)	Adjusted (n = 325)†
NCS use at study entry	1.51 (0.61-3.70), *P* = .4	1.60 (0.62-4.11), *P* = .3

*Data presented as HR (95% CI) and *P* value. The total sample size with available data included in the model (n) is also shown. Estimates obtained from unadjusted and adjusted Cox regression models.

†The adjusted model includes age (as a nonlinear term with restricted cubic splines), sex, race and ethnicity (categorized as non-Hispanic White vs other), and body mass index percentile as covariates.

ARTICLE IN PRESS

TABLE E4. The association of using medications for allergic rhinitis other than NCS at study entry with the risk of SARS-CoV-2 infection among adults with a prior health care provider diagnosis of allergic rhinitis*

	Unadjusted (n = 932)	Adjusted (n = 906)†
Use of medications for allergic rhinitis other than NCS at study entry	0.93 (0.55-1.55), *P* = .8	0.98 (0.58-1.65), *P* = .9

*Data presented as HR (95% CI) and *P* value. The total sample size with available data included in the model (n) is also shown. Estimates obtained from unadjusted and adjusted Cox regression models.

†The adjusted model includes age (as a nonlinear term with restricted cubic splines), sex, race and ethnicity (categorized as non-Hispanic White vs other), and body mass index percentile as covariates.

TABLE E5. The association of the number of allergic rhinitis medications used at study entry with the risk of SARS-CoV-2 infection among adults with a prior health care provider diagnosis of allergic rhinitis*

	Unadjusted (n = 932)	Adjusted (n = 906)†
Number of allergic rhinitis medications used at study entry	1.16 (0.87-1.56), *P* = .3	1.19 (0.89-1.60), *P* = .2

*Data presented as HR (95% CI) and *P* value. The total sample size with available data included in the model (n) is also shown. Estimates obtained from unadjusted and adjusted Cox regression models.

†The adjusted model includes age (as a nonlinear term with restricted cubic splines), sex, race and ethnicity (categorized as non-Hispanic White vs other), and body mass index percentile as covariates.

TABLE E6. The association of ICS use at study entry with the risk of COVID-19 among children ages 5 years and older with a prior health care provider diagnosis of asthma*

	Unadjusted (n = 679)	Adjusted (n = 609)†
ICS use at study entry	0.66 (0.31-1.41), *P* = .3	0.81 (0.36-1.80), *P* = .6

*Data presented as HR (95% CI) and *P* value. The total sample size with available data included in the model (n) is also shown. Estimates obtained from unadjusted and adjusted Cox regression models.

†The adjusted model includes age (as a nonlinear term with restricted cubic splines), sex, race and ethnicity (categorized as non-Hispanic White vs other), and body mass index percentile as covariates.

TABLE E7. The association of ICS use at study entry and end of study with the risk of COVID-19 among adults with a prior health care provider diagnosis of asthma*

	Unadjusted (n = 292)	Adjusted (n = 282)†
ICS use at study entry and end of study	1.80 (0.76-4.30), *P* = .2	2.20 (0.95-5.10), *P* = .07

*Data presented as HR (95% CI) and *P* value. The total sample size with available data included in the model (n) is also shown. Estimates obtained from unadjusted and adjusted Cox regression models.

†The adjusted model includes age (as a nonlinear term with restricted cubic splines), sex, race and ethnicity (categorized as non-Hispanic White vs other), and body mass index percentile as covariates.

TABLE E8. The association of using asthma controllers other than ICS at study entry and with the risk of SARS-CoV-2 infection among adults with a prior health care provider diagnosis of asthma*

	Unadjusted (n = 441)	Adjusted (n = 427)†
Use of asthma controllers other than ICS at study entry	1.71 (0.67-4.40), *P* = .3	2.02 (0.80-5.11), *P* = .1

*Data presented as hazard ratio (95% CI) and *P* value. The total sample size with available data included in the model (n) is also shown. Estimates obtained from unadjusted and adjusted Cox regression models.

†Adjusted model includes age (as a nonlinear term with restricted cubic splines), sex, race and ethnicity (categorized as non-Hispanic White vs other), and body mass index percentile as covariates.

TABLE E9. The association of the number of asthma controllers used at study entry with the risk of SARS-CoV-2 infection among adults with a prior health care provider diagnosis of asthma*

	Unadjusted (n = 441)	Adjusted (n = 427)†
Number of asthma controllers used at study entry	1.56 (0.91-2.67), P = .1	1.82 (1.07-3.08), P = .03

*Data presented as HR (95% CI) and P value. The total sample size with available data included in the model (n) is also shown. Estimates obtained from unadjusted and adjusted Cox regression models.

†The adjusted model includes age (as a nonlinear term with restricted cubic splines), sex, race and ethnicity (categorized as non-Hispanic White vs other), and body mass index percentile as covariates.

TABLE E10. The association of NCS and/or ICS use at study entry with the risk of SARS-CoV-2 infection among adults with a prior health care provider diagnosis of allergic rhinitis or asthma*

	Unadjusted (n = 1047)	Adjusted (n = 1017)†
NCS and/or ICS use at study entry:	Reference	Reference
Not using NCS or ICS	1.43 (0.56-3.70)	1.44 (0.54-3.87)
Using only ICS	1.70 (1.01-2.90)	1.77 (1.05-2.98)
Using only NCS	2.04 (0.82-5.10)	2.21 (0.90-5.43)
Using both NCS and ICS	*P* = .2	*P* = .1

*Data presented as HR (95% CI). The term *P* value and total sample size with available data included in the model (n) are also shown. Estimates obtained from unadjusted and adjusted Cox regression models.

†The adjusted model includes age (as a nonlinear term with restricted cubic splines), sex, race and ethnicity (categorized as non-Hispanic White vs other), and body mass index percentile as covariates.
